# A Case Report on Managing Hemolysis, Elevated Liver Enzymes, and Low Platelets (HELLP Syndrome) During a Rapid Response Code

**DOI:** 10.7759/cureus.11028

**Published:** 2020-10-18

**Authors:** Nida Hasan, Margaret McGrath, Gillean Cortes, Jacob Miller, Charles Deck

**Affiliations:** 1 Obstetrics and Gynecology, A.T. Still University School of Osteopathic Medicine in Arizona, Mesa, USA; 2 Medicine, A.T. Still University School of Osteopathic Medicine in Arizona, Mesa, USA; 3 Internal Medicine, The Wright Center for Graduate Medical Education, Scranton, USA

**Keywords:** hellp syndrome, rapid response

## Abstract

HELLP syndrome (hemolysis, elevated liver enzymes, and low platelets) is a relatively rare condition that can complicate pregnancies. We present a 31-year-old gravida 5 para 0 female at 37-3/7 weeks gestation who presented with sudden onset severe epigastric pain, shortness of breath, diaphoresis, bradycardia, and elevated blood pressure. A Rapid Response Team (RRT) was called, and the patient was treated with IV magnesium in addition to blood pressure control medications. Laboratory results confirmed the diagnosis of HELLP syndrome. HELLP syndrome has the potential for major thrombotic complications and should be considered. Early consideration and evaluation of thrombotic complications amongst other common diagnoses are important for prompt treatment and optimization of maternal and fetal well-being.

## Introduction

HELLP syndrome (hemolysis, elevated liver enzymes, and low platelets) is a relatively rare condition that can complicate pregnancies [1]. It occurs in 0.5 to 0.9% of pregnancies and in 10-20% of cases with severe preeclampsia. HELLP syndrome is sometimes considered, controversially, as a severe form of preeclampsia however some patients who present with HELLP do not have prior hypertension or proteinuria [2,3]. Most often, HELLP syndrome occurs in the third trimester [4].

Common symptoms include abdominal pain, nausea, and vomiting as well as non-specific symptoms related to preeclampsia. Diagnosis is dependent on certain criteria and can be misdiagnosed due to the wide differential including fatty liver of pregnancy, hepatitis, cholecystitis, thrombocytopenia, or other microangiopathic hemolytic disorders [4]. While there are various etiologies of HELLP syndrome, cardiopulmonary presentations may make it more challenging to reach a diagnosis and subsequent management. This case highlights the presentation of HELLP syndrome in a patient following a rapid response code.

## Case presentation

We present a 31-year-old gravida 5 para 0 female at 37-3/7 weeks gestation without a significant past medical history or previous complications during this pregnancy. She presented to the emergency room for sudden onset severe epigastric pain while driving home from work, severely short of breath, diaphoretic, and bradycardic with an initial blood pressure of 214/105. A Rapid Response Team (RRT) was called and the patient was treated with IV magnesium in addition to IV hydralazine/labetalol. Systolic blood pressure readings decreased to the 160-180 mm Hg range. The patient initially appeared lethargic but did not lose consciousness; she had mild rigors without seizure activity and was found to be hypothermic to 94.1℉ at one point, for which a heating blanket was applied.

**Figure 1:**
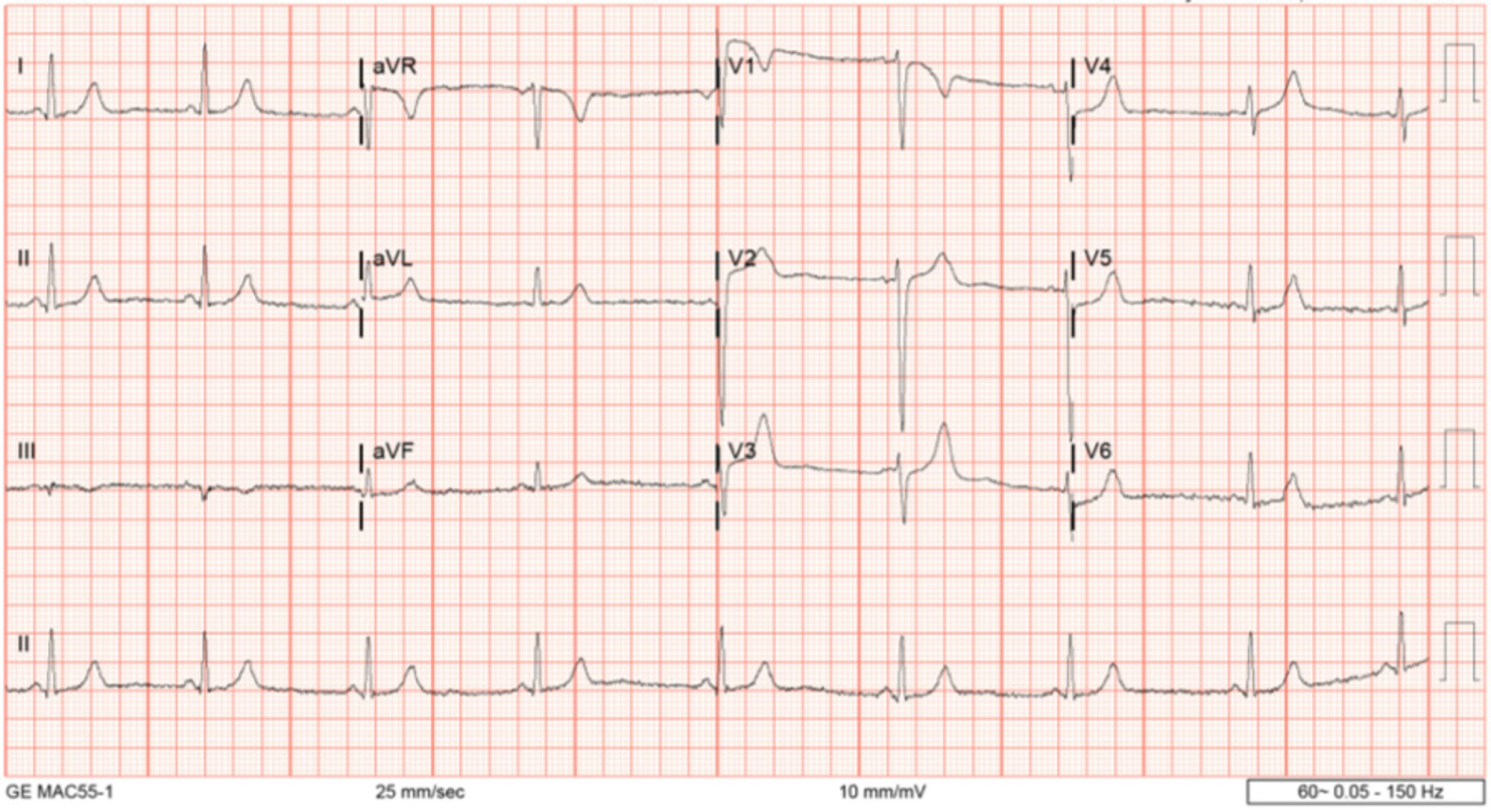
Initial EKG showing sinus bradycardia with slightly prominent T waves, but no ST depression or elevation EKG: electrocardiogram

Laboratory values were significant for elevated transaminases (alanine aminotransferase (ALT): 694 units/L (U/L); aspartate aminotransferase (AST): 1028 U/L)), decreased platelet counts (72 x 10^9^/L), and elevated lactic acid dehydrogenase (LDH) (2082 U/L) consistent with HELLP syndrome, for which she was admitted. Initial electrocardiogram (EKG) showed sinus bradycardia with slightly prominent T waves, but no ST depression or elevation, and no ventricular ectopy (Figure 1). 

Troponins were ordered to rule out acute coronary syndrome. Fetal monitoring was maintained throughout the RRT. Venous Doppler studies of the lower extremities showed sluggish flow but no evidence for acute deep vein thrombosis (DVT). The patient was subsequently taken for emergency low transverse Cesarean section (LTCS) which was performed without incident and transferred to the ICU postoperatively for close monitoring. 

Postoperatively, she complained of some expected incisional pain but denied lower rib cage or upper abdominal pain. She continued on combination IV magnesium/oxytocin, with blood pressure readings in the 130s-150s/90s-100s range; an additional dose of IV labetalol was given with improvement in blood pressure readings. She denied headaches, blurred vision, double vision, substernal chest pain, palpitations, increased shortness of breath, pleurisy, or recurrent nausea/vomiting. 

She remained in the ICU for four days postoperatively. All labs continued to trend towards normal, including her blood pressure. Her platelets improved to 117 x 10^9^/L, and liver function tests (LFTs) were slightly elevated, though trending towards normal (AST 38 U/L, ALT 147 U/L). She was discharged with nifedipine and labetalol for blood pressure control. Her discharge precautions were to return if she experienced severe headaches, dizzy spells, blurry vision, chest pain, upper abdominal pain, or blood pressures over 140/90. The patient reported significant improvement in her clinical symptoms, continued to meet her postoperative milestones, and was discharged with a follow-up visit scheduled in one week for blood pressure monitoring and staple removal.

## Discussion

HELLP syndrome is defined by hemolysis, elevated liver enzymes, and thrombocytopenia [1]. It occurs in 0.5 to 0.9% of pregnancies, most often in the third trimester or in the few days immediately postpartum [4]. The patient met the criteria for HELLP syndrome as defined by the Tennessee Classification System, which places strict parameters for complete HELLP syndrome: intravascular hemolysis defined by either abnormal peripheral blood smear or by increased serum bilirubin (≥1.2 mg/100 mL) and elevated LDH (>600 U/L), elevated LFTs defined as AST or ALT greater than or equal to two times the upper level of normal, and low platelets defined as ≤100 x 10^9^/L.

The common differential diagnoses for HELLP syndrome include fatty liver of pregnancy, thrombotic thrombocytopenic purpura, pregnancy-related hemolytic uremic syndrome, and systemic lupus erythematosus. However, other severe complications such as cardiopulmonary conditions need to be considered when patients present with symptoms of HELLP as in this patient. She additionally presented with respiratory distress, diaphoresis, and bradycardia which can be concerning for a pregnant patient at full-term. Therefore, it is practical to rule out such conditions as ST-elevation myocardial infarction (STEMI), pulmonary embolism (PE), or DVT in patients with HELLP syndrome. 

There are a few cases in published literature associating HELLP syndrome and postpartum cardiomyopathy. In one report, a case of HELLP syndrome presented as non-STEMI while another two cases presented as acute coronary syndrome [1]. Another study showed factor V Leiden was more prevalent in a group of patients with HELLP syndrome compared to controls [5]. HELLP syndrome is considered a microangiopathic hemolytic disorder and thus has the potential for major thrombotic complications [6]. We believe this case highlights the importance of considering the relationship between hypertensive disorders of pregnancies and thrombophilias when evaluating a patient with HELLP syndrome. 

Abdominal pain in a pregnant patient can also be concerning for other disorders. The Society for Maternal-Fetal Medicine recommends the consideration of amniotic fluid embolism in the differential diagnosis of sudden cardiorespiratory collapse in pregnant patients [7]. Amniotic fluid embolism is a rare, potentially fatal condition that can present with hypoxia, hypotension, or hemodynamic collapse, and coagulopathy [8]. Treatment involves aggressive supportive care and may require cardiopulmonary resuscitation along with vascular management. Appendicitis, diverticulitis, and ovarian cysts are other more common diagnoses to consider. Placenta accreta spectrum can lead to significant maternal morbidity and mortality. A devastating complication is severe hemorrhage and possibly disseminated intravascular coagulation [9,10]. Premature birth may occur [11]. High clinical suspicion is warranted in a patient presenting with HELLP syndrome as other uncommon diagnoses may be missed and management may be misguided. As in this patient, management includes possible immediate delivery to improve maternal and fetal survival [12].

## Conclusions

HELLP syndrome is a rare condition that most commonly presents in pregnant patients with abdominal pain. However, it may present with other non-specific symptoms such as respiratory distress, as seen in our patient, which may further complicate the pregnancy and diagnosis. In conclusion, it is important to consider cardiopulmonary conditions such as STEMI and DVT in patients amongst other diagnoses with HELLP syndrome in correlation to their clinical picture. This can help predict and prompt early treatment and further management.
